# Barriers and facilitators to the quality use of essential medicines for maternal health in low–resource countries: An Ishikawa framework

**DOI:** 10.7189/jogh.05.010406

**Published:** 2015-06

**Authors:** Dan N. Tran, Lisa A. Bero

**Affiliations:** 1 Department of Clinical Pharmacy, University of California, San Francisco School of Pharmacy, CA, USA; 2 Institute for Health Policy Studies, University of California, San Francisco, CA, USA; 3 World Health Organization Collaborating Centre on Pharmaceutical Research and Science Policy, University of California, San Francisco, CA, USA; 4 Charles Perkins Centre, Faculty of Pharmacy, University of Sydney, Sydney, Australia

## Abstract

**Background:**

An estimated 800 women die every day due to complications related to pregnancy or childbirth. Complications such as postpartum haemorrhage (PPH) and pre–eclampsia and eclampsia can be prevented by the appropriate use of essential medicines. The objective of this study was to identify the common barriers and facilitators to the availability and use of oxytocin, ergometrine, and magnesium sulfate (MgSO_4_) – essential medicines indicated for the prevention and treatment of PPH and pre–eclampsia and eclampsia.

**Methods:**

We analyzed seven UNFPA/WHO reports published in 2008–2010. These reports summarized country–wide rapid assessments of access to and use of essential medicines for maternal health in Mongolia, Nepal, Laos, the Democratic People’s Republic of Korea (DPRK), the Philippines, Vanuatu, and the Solomon Islands. We used a “fishbone” (Ishikawa) diagram as the analytic framework to identify facilitators and barriers at four health–system levels: government/regulatory, pharmaceutical supply, health facility, and health professional.

**Results:**

Common facilitators to the quality use of essential medicines for maternal health were observed at the government/regulatory and health professional level. A majority of countries had these medicines listed in their essential medicines lists. Awareness of the medicines was generally high among health professionals. Common barriers were identified at all health–system levels. First, standard treatment guidelines were not available, updated, or standardized. Second, there was an inadequate capacity to forecast and procure medicines. Third, a required MgSO_4_ antidote was often not available and the storage conditions for oxytocin were deficient.

**Conclusions:**

The “fishbone” Ishikawa diagram is a useful tool for describing the findings of rapid assessments of quality use of essential medicines for maternal health across countries. The facilitators and barriers identified should guide the development of tailored intervention programs to improve and expand the use of these life–saving medicines.

Approximately 800 women die every day due to complications during pregnancy or childbirth [1]. An overwhelming 99% of these maternal deaths occur in low–resource settings, with Sub–Saharan Africa and Southern Asia accounting for 86% of overall global maternal mortality cases in 2013 [1,2]. Moreover, the probability that a 15–year–old woman will eventually die from a cause related to maternal health is much higher for women living in low income countries than for those who live in high income countries (1:160 vs 1:3700) [2,3]. The higher number of pregnancies on average and a higher risk associated with each birth contribute to the higher adult lifetime risk of maternal death [3].

In 2000, 189 member states of the United Nations adopted eight Millennium Development Goals (MDG) [4]. The fifth MDG aims to reduce maternal mortality worldwide by 75% between 1990 and 2015 [4,5]. Despite a 45% decrease in maternal mortality in the past two decades, the annual rate of decline has been far below the MDG 5 target [2,5,6]. A lack of sufficient antenatal care during pregnancy and inadequate assistance from skilled health providers during delivery contribute to the high maternal mortality rate in developing countries [7]. The World Health Organization (WHO) reports that between 2003 and 2009, more than half of all maternal deaths resulted from haemorrhage (with postpartum haemorrhage (PPH) accounting for more than two thirds of cases), hypertensive disorders (pre–eclampsia and eclampsia), sepsis, and unsafe abortion [8]. WHO has provided evidence–based recommendations for the essential interventions and medicines needed to improve maternal health and prevent these maternal complications [9–12]. Even though the availability of essential medicines for maternal health is not well documented in many countries [13], recent data suggested that it is low in Africa and Asia [14].

To investigate the availability and use of WHO–recommended life–saving medicines for women and children, WHO and the United Nations Population Fund (UNFPA) conducted a descriptive study of essential medications for maternal, child, and reproductive health in seven low–resource countries between 2008 to 2010. The two objectives of our study were: (1) to obtain a “snapshot” of the availability of oxytocin, ergometrine, and magnesium sulfate (MgSO_4_), and (2) to use the Ishikawa “fishbone” diagram as a framework to describe the common barriers and facilitators contributing to use of these essential medicines.

## METHODS

Our primary data sources were the published UNFPA/WHO reports of field assessments conducted in seven countries between September 2008 and November 2010 (available from the authors). The reports summarized country–wide rapid assessments of access to and use of essential medicines for maternal and newborn health care and reproductive health.

### Selection of study sites

Seven low–resource countries located in Asia and the Pacific Ocean were included in the study: Mongolia, Nepal, Laos, DPRK, the Philippines, Vanuatu, and the Solomon Islands. In each country, health care facilities (ie, nurse aid posts, health units/centers/clinics, hospitals) and medicine–supply facilities (ie, medical warehouse and pharmacies) from multiple sectors were purposively selected based on the site’s population density, site’s performance on MDG 5, transportation feasibility, human capacity, and time constraints. The sample included facilities that provided various levels of care (primary, secondary, and tertiary), from different governmental sectors (central, provincial, district, and below), and with different types of financial support (publicly–, privately–, or non–governmental organization–funded).

### Selection of medicines

The medicines evaluated were on the WHO Model List of Essential Medicines and some were listed as priority life–saving medicines for women and children by the WHO Department of Essential Medicines and Health Products [11,12]. These medicines included: oxytocin and ergometrine injections for prevention and treatment of PPH; MgSO_4_ injection for prevention and treatment of severe pre–eclampsia and eclampsia; ampicillin, gentamicin and metronidazole injections for treatment of maternal sepsis; ampicillin, gentamicin, procaine benzylpenicillin, and ceftriaxone for neonatal sepsis; and contraceptives including oral, emergency, injectable, and implant formulations. In this study, we focused on three medicines – oxytocin, ergometrine, and MgSO_4_ – for which data were available and consistently reported for all seven countries. We also collected information pertaining to the availability of calcium gluconate – the recommended antidote for MgSO_4_ toxicity – whenever the relevant data were reported.

### Data

The data in each country report consisted of observations, interviews and archival analysis. For each country, a collaborative team of researchers from WHO, UNFPA, the Ministry of Health (MoH), and local representatives conducted site visits. Interviews were conducted with local partners and stakeholders such as representatives from the MoH, professional organizations, pharmaceutical administration authorities, reproductive and maternal health non–governmental organizations (NGOs). Documents relevant to the use of medicines were also reviewed. Examples of relevant documents included, but were not limited to, national essential medicine lists, standard treatment guidelines and protocols, training manuals, procurement policies and reports, commodity security status assessment reports, and national health strategic plans.

The following data were collected for each medicine: (1) need and demand, (2) availability, (3) presence on essential medicine lists, (4) inclusion in standard treatment guidelines and protocols, (5) rational use, (6) licensing and areas of quality assurance, (7) storage, (8) procurement and supply chain, (9) costs, and (10) coordination and integration between public and private collaboration efforts.

### Fishbone (Ishikawa) diagram as the analytic framework and assessment tool

The Ishikawa diagram is also known as the “fishbone” or “cause and effect” diagram. It was developed by Kaoru Ishikawa in 1968 and is well known in the quality management, quality control, and manufacturing industry [15]. The Ishikawa diagram shows a visual representation of potential causes contributing to an overall outcome. Recently, it has been used as an analytic tool in the health sciences field. In 2010, Ridge et al. proposed the development and use of this diagram to rapidly assess the barriers and facilitators to the availability and use of MgSO_4_ in Zambia [16]. In 2013, Bigdeli et al. used this diagram as the conceptual framework to identify health system barriers to access and use of MgSO_4_ in Pakistan [17].

In this study, we used the fishbone Ishikawa diagram that Ridge et al. developed to assess the barriers and facilitators to quality use of three essential medicines – oxytocin, ergometrine, and MgSO_4_. For each medication, Ishikawa diagrams of facilitators and barriers were created and modified using an iterative process based on the data extracted from each country report.

The four health system levels influencing barriers and facilitators to the quality use of essential medications were defined as: (1) government/regulatory, (2) pharmaceutical supply system, (3) health facility, and (4) and health professional [16]. We extracted data from the country reports that were specific to these four levels. At the government/regulatory level, we collected medication–specific information regarding inclusion in essential medicine lists, medicine licensure, and recommendations in national and local standard treatment guidelines. At the pharmaceutical supply system level, we extracted information regarding procurement and supply procedures for each medication. At the health facility level, we collected data regarding access to care and the equipment and supplies necessary for diagnosing procedures and drug storage. Lastly, at the health professional level, we extracted data associated with health providers’ knowledge and practice. All extracted textual data relevant to each of the four health–system levels proposed in our analytic framework were recorded in Excel.

We interpreted and fitted the extracted data from the seven reports into the appropriate categories in the Ishikawa diagram. **Table 1** shows the categories that were used to summarize extracted data.

**Table 1:** Components required for the quality use of an essential medicine by health system level

Government/regulatory level	Pharmaceutical supply system level	Health facility level	Health professional level
● Included in the essential medicine list ● Quality–assured medicine licensed for use in country ● Recommended treatment in national standard treatment guideline ● Standard treatment guideline translated into suitable local protocol	● Suitable procurement procedure in place ● Medicine supplied to health care facility	● Woman have access to care (antenatal care or skilled birth attendants) ● Equipment and supplies available for diagnosis of complications and drug storage ● Correct diagnoses are made	● Health providers aware medicine is first–line treatment ● Staff trained to use medicine ● Trained staff available to administer medicine ● Equipment and supplies are available to administer medicine

### Availability of medicines

Each report recorded the number of health care centers and medicine–supply facilities visited, as well as whether each of the three medicines was available at each facility on the day of visit. We calculated the availability of an individual medicine as the percentage of facilities where each medicine was reported as available on the day of data collection. Because its availability and timely administration is crucial to reverse MgSO_4_ toxicity, we also calculated the availability of calcium gluconate [18].

### Data presentation

We present the availability data and Ishikawa diagrams for each medicine. We provide a qualitative summary of the facilitators and barriers derived from the Ishikawa diagrams.

### Ethical approval

Because this study was a secondary analysis of published reports, institutional review board (IRB) approval was not required.

## RESULTS

### Availability

Availability of the three medicines varied by country and medicine (**Table 2**). Oxytocin had high availability in six out of seven countries compared to ergometrine. The two exceptions were the Philippines and the Solomon Islands, where ergometrine was available in a higher percentage of facilities than oxytocin. Four countries had MgSO_4_ available at less than 60% of their facilities, with only 18% of health facilities carrying MgSO_4_ in Laos. Calcium gluconate, a required antidote for MgSO_4_ toxicity, was not consistently available when MgSO_4_ was present.

**Table 2:** Availability of medicines by country

	Availability of medicine (% of facilities)†						
	**Laos (n = 34)**	**Mongolia (n = 39)**	**Nepal (n = 26)**	**DPRK (n = 11)**	**Philippines (n = 40)**	**Vanuatu (n = 6)**	**Solomon Islands (n = 16)**
**Medicine**							
**Oxytocin**	50	85	89	73	70	100	69
Ergometrine*	n/r	51	54	n/r	73	83	88
**MgSO_4_**	18	95	58	82	55	33	86
**Calcium gluconate**	n/r	69	39	n/r	35	0	72

DPRK – Democratic People's Republic of Korea, n/r – not reported

*Syntometrine (combination of oxytocin and ergometrine) on Solomon Islands.

†The availability of an individual medicine was calculated as the percentage (%) of facilities where each medicine was reported as available on the day of data collection.

### Facilitators and barriers to quality use of medicines

**Figure 1:**
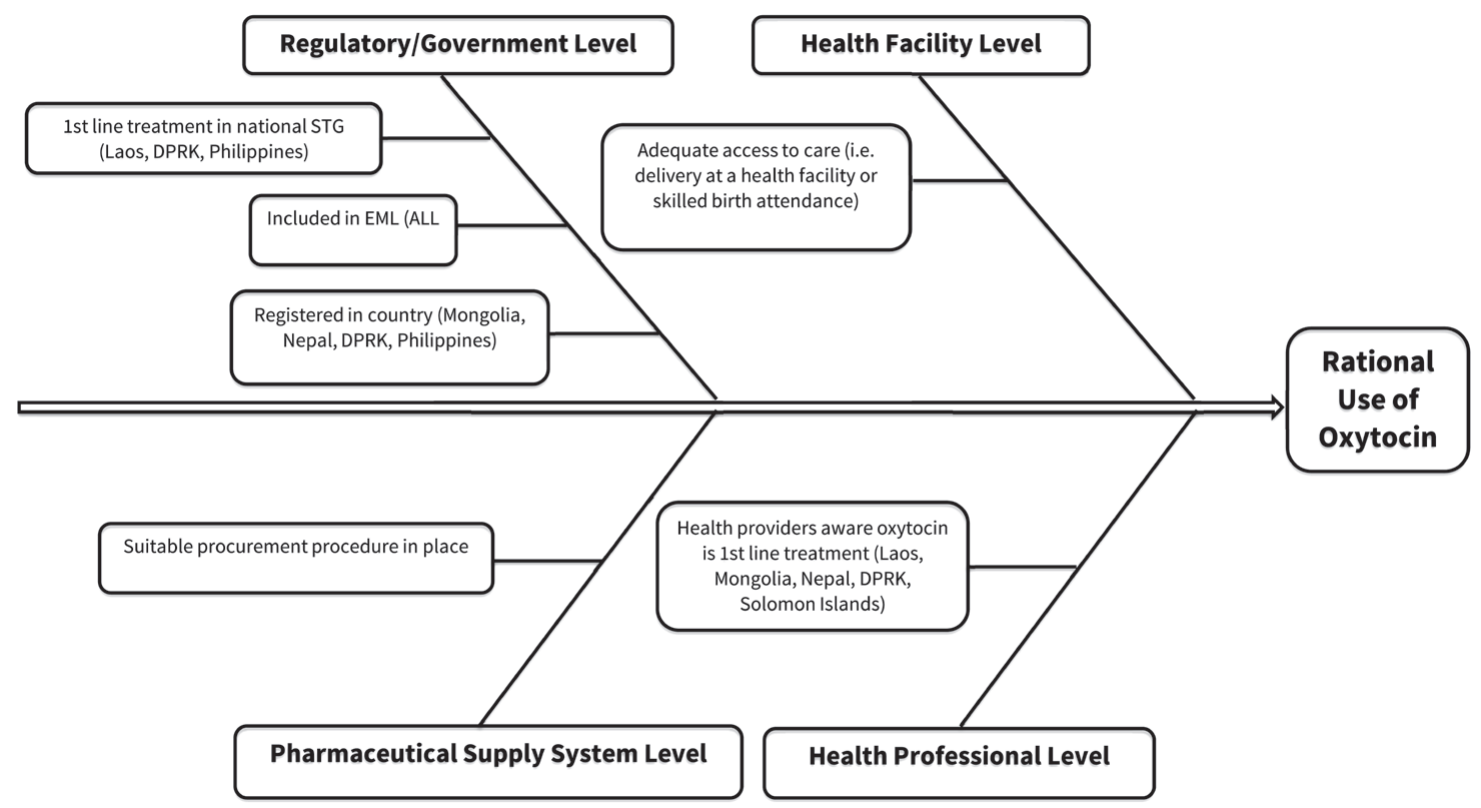
Facilitators to the availability and use of oxytocin.

**Figure 2:**
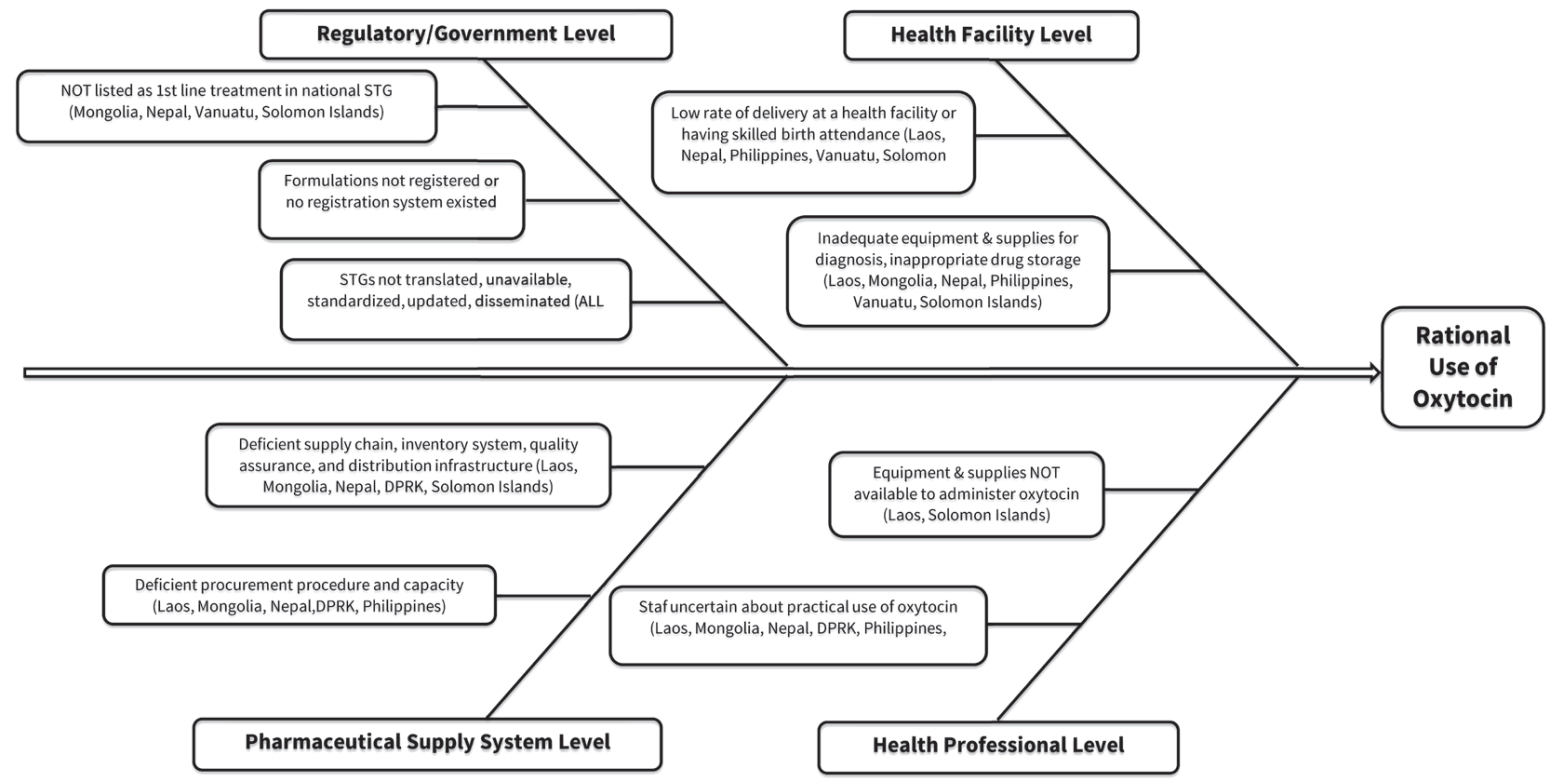
Barriers to the availability and use of oxytocin.

**Figures 1** and **2** show the fishbone diagrams of the facilitators and barriers for oxytocin as examples. Fishbone diagrams for all medicines are available in the **Online Supplementary Document(Online Supplementary Document)**. **Table 3** summarizes the analysis from the fishbone diagrams and shows the facilitators and barriers to the availability and use of oxytocin, ergometrine, and MgSO_4_ across all four health–system levels. The section below describes the barriers and facilitators at each level of analysis: regulatory/government, pharmaceutical supply system, health facility and health professional.

**Table 3:** Summary of facilitators and barriers to the availability and use of oxytocin, ergometrine, and MgSO_4_

Health system level	Facilitators	Barriers
**Government/Regulatory**	Essential medicine included in the national EML	● STGs for pre–eclampsia/eclampsia and PPH prevention and treatment were not consistent, updated, or disseminated ● Ergometrine and syntometrine recommended as first–line for PPH prevention and treatment ● Formulations not licensed by national drug authority ● No drug registration system
**Pharmaceutical supply System**	Essential medicine listed on the national EML	● Lack of adequate and suitable procurement and forecasting system in place ● No stringent quality assurance process, especially storage conditions to maintain drug efficacy ● Inadequate infrastructure led to stock–outs in health facilities
**Health facility**	Essential medicines found in health facilities	● Wide variation in the level of availability between different countries ● Lack of stringent requirements for maintenance of equipment used to store medications ● Lack of adequate diagnostic testing equipment to make correct diagnosis
**Health professional**	Health professionals aware of recommended first–line medicines for PPH and pre–eclampsia/eclampsia prevention and treatment	● Uncertainties in the practical administration of essential medications ● Lack of continuous professional education for health providers ● Lack of equipment to safely administer medications

#### 1. Regulatory/Government Level

**Oxytocin.** Oxytocin is the WHO recommended uterotonic drug for the prevention and treatment of PPH [10,12]. Oxytocin was included in the essential medicine lists (EML) of all seven countries. Indications for use were included in the EMLs of six countries, with the exception of the Philippines. A functional drug registration system in compliance with WHO–Good Manufacturing Practices guideline existed in all countries except the Solomon Islands. Data for oxytocin licensing status was not consistently reported across all seven countries. However, none of the oxytocin formulations were licensed in Laos. Standard treatment guidelines recommended oxytocin as a first line medicine for prevention of treatment of PPH in Laos, DPRK, and the Philippines. In contrast, Vanuatu and the Solomon Islands recommended ergometrine or syntometrine (combination of oxytocin and ergometrine) as a first–line drug treatment, which was not consistent with WHO evidence–based recommendations. Across all seven countries, standard treatment guidelines were inconsistent, out–of–date, and not widely disseminated.

**Ergometrine.** Ergometrine is the second–line recommended uterotonic drug for prevention and treatment of PPH when oxytocin is unavailable or when bleeding does not respond to oxytocin [10]. The use of ergometrine is limited by its side effects and contraindication in patients who have high blood pressure. In Mongolia and the Solomon Islands, ergometrine was not listed on the national EMLs. Ergometrine was licensed for use in three countries (Nepal, DPRK, the Philippines). Ergometrine and syntometrine were recommended as first–line drugs for PPH in Vanuatu and on the Solomon Islands, respectively. Standard treatment guidelines for prevention and treatment of PPH and the use of ergometrine were unavailable or not updated according to the most current WHO clinical guidelines.

**MgSO_4_.** MgSO_4_ **i**njection is recommended for the prevention and treatment of eclampsia in women with severe pre–eclampsia [9,12]. MgSO_4_ was included as an essential medicine in the national EMLs of all seven countries. Indications were clearly provided with the exception of the Philippines. In Laos, the recommended formulation (50% solution) was not found to be licensed or available; 20% and 15% formulations were observed in facilities. DPRK was the only country which reported translating and utilizing treatment guidelines for pre–eclampsia and eclampsia in partnership with WHO, UNFPA, and other national professional associations. Overall, at health facilities, there was a lack of treatment guidelines, treatment protocols, and educational materials for the management of this pregnancy complication.

#### 2. Pharmaceutical supply system level

The reports identified commonalities across countries regarding the procurement of oxytocin, ergometrine, and MgSO_4_. First, procurement of drugs was based on each country’s EML. Second, there was a lack of accurate, consistent, and scientific methods to estimate and forecast the use of medicines. Last, there was a lack of resources and capacity to optimize the procurement process. Package inserts or drug labels provided inadequate, non–specific information, and were sometimes written in languages not widely understood in the country, such as English, Russian, Thai, or Chinese. Additionally, a majority of countries reported an inadequate supply chain due to inconsistency in inventory (ie, stock card management), delay in supply leading to stock–outs (ie, from agencies that donated essential medicines), or an inadequate infrastructure and lack of transportation to health facilities. Vanuatu was the only country found to have a well–integrated procurement mechanism and supply system for essential medicines. Structured and standardized forms and drug order lists were available for use both in public and NGO facilities.

#### 3. Health Facility Level

**Oxytocin.** Giving birth in an environment where trained health providers such as doctors, nurses, or midwives are available (skilled birth attendants) can help provide timely medical interventions to prevent complications and death. A majority of the countries reported having 15% to 38% of births delivered without skilled birth attendants. In Laos and Nepal, a high percentage of births took place at home, 85% and 81% respectively. In the Philippines, a higher number of deliveries with skilled birth attendants occurred in urban vs rural areas. On the Solomon Islands, about 15% of births were assisted by nurse aides with the least amount of in–service training.

Additionally, Laos and the Solomon Islands reported a lack of diagnostic equipment for PPH. Expired drugs and stock–outs were observed in health facilities in Vanuatu and the Philippines.

The storage condition of oxytocin was widely reported as inappropriate. Oxytocin is a heat–sensitive medicine and must be kept between 2–8°C. Reproductive health kits, which contained oxytocin, were stored at room temperature. In addition, the cold chain requirements for this medication were at risk due to unstable power supply or inadequate maintenance of refrigerators.

**Ergometrine.** Results pertaining to skilled birth attendance and availability of diagnostic equipment were similar to findings reported for oxytocin. Ergometrine also has restricted storage conditions. It is a light–sensitive medication, but was stored inside the delivery room, unpacked, and exposed to sunlight.

**MgSO_4_.** Receiving antenatal care allows for early detection, management, and prevention of hypertension during and after pregnancy. The percentages of women who did not receive antenatal care ranged from 75% in Laos to 16% in Vanuatu. Antenatal care was provided to 99% of women in DPRK and 95% on the Solomon Islands. In Laos, several health centers lacked adequate equipment to do urine and haemoglobin testing in order to make correct diagnosis for patients with severe pre–eclampsia. The lack of diagnostic equipment as well as equipment to administer MgSO_4_ safely was also reported in primary health centers on the Solomon Islands, even though a high percentage of health facilities (86%) had the medication in stock. Calcium gluconate, a required antidote for MgSO_4_ toxicity, was not available in many facilities across all seven countries.

#### 4. Health professional level

**Oxytocin.** There was a relatively high level of awareness of the need to use oxytocin for every delivery as part of the active management of third stage of labor (AMTSL) in most countries assessed. However, other treatments were sometimes preferred over the use of oxytocin (ie, ergometrine, syntometrinecarbazochrome). There were also uncertainties as to when to administer the drug, what the maximum dose was, and whether it should be administered in combination with ergometrine. Health professionals were unaware of the correct storage conditions for the medication.

**Ergometrine.** Ergometrine alone or ergometrine/oxytocin combination was recognized as the first–line medication for prevention and treatment of PPH in Vanuatu and the Solomon Islands, even when WHO recommendations for prevention and treatment of PPH stated that oxytocin should be the drug–of–choice uterotonic agent.

**MgSO_4_.** Health professional awareness of the use of MgSO_4_ was adequate in Laos, Mongolia, and Nepal. Nevertheless, in Mongolia, Vanuatu, and the Solomon Islands there was uncertainty about how MgSO_4_ should be administered. Furthermore, a lack of equipment to safely administer MgSO_4_ was seen in Laos and the Solomon Islands.

## DISCUSSION

The “fishbone” Ishikawa diagram was used as the analytic framework to describe the findings of rapid assessments of essential medicines for maternal health conducted in seven countries. We identified common facilitators and barriers to the availability and use of oxytocin, ergometrine, and MgSO_4_ across 4 health system levels: (1) government/regulatory, (2) supply system, (3) health facility, and (4) and health professional.

### Facilitators

The first common facilitator at the government policy level was that all three essential medicines were consistently listed on national EMLs. This was an encouraging finding as oxytocin, ergometrine, and MgSO_4_ were listed on the WHO EML [11]. The WHO Model List of Essential Medicines is developed based on the following criteria: prevalence of diseases, efficacy and safety of treatment recommendations, and comparative cost–effectiveness analysis [19]. In the past three decades, it has been increasingly used within national health systems to ensure adequate supply, appropriate dosages, formulations, and indications. Adapting the WHO EML into a national EML is highly encouraged. In fact, it has been argued that having a functional EML is a “strong indicator” of an effective health system since it provides guidance for adequate procurement and supplies of medicines in a particular country [20]. National EMLs should therefore be more integrated into procurement procedures in each country to ensure consistent supply of essential medicines at all time.

Our second common facilitator was identified at the health professional level. We found that there was generally high knowledge, awareness, and acceptance of essential medicines as first–line treatment options for PPH and pre–eclampsia/eclampsia. Knowledge of a medicine is a necessary prerequisite to using it [21]. Additionally, a high awareness and acceptance of the use of essential medicines, especially by local opinion leaders can promote evidence–based practice and facilitate the adoption of clinical practice guidelines [22].

### Barriers

We identified major barriers to use of the medicines in each health–system level evaluated, particularly in areas related to standard treatment guidelines, drug procurement, drug supply and storage, and staff training.

First, it was consistently reported that there was a lack of local standard treatment guidelines for the management of pre–eclampsia/eclampsia and active management of the third stage of labor. The lack of guidelines could reasonably be accounted for by the lack of translation of WHO treatment guidelines into suitable local guidelines or by a lack of an adequate dissemination mechanism for guidelines and their derivative products, such as teaching materials, posters, and visible treatment flowcharts. A positive example of the successful translation of guidelines to suitable local protocols was observed in DPRK. In this country, WHO guidelines for maternal and child health were translated and printed. The content of derivative products, such as posters, was consistent with WHO guidelines. This finding shows that collaboration with international organizations such as WHO and UNFPA to promote the use of suitable standardized treatment guidelines should be encouraged in order to assist health care providers in their treatment decision–making process.

Second, a suitable procurement process was generally not in place due to inadequate capacity for forecasting essential medicines or a lack of documentation of procurement procedures. However, there was evidence that a few countries, such as Vanuatu and the Solomon Islands, had better stock maintenance, inventories, and ordering systems in place. Training programs for personnel that focus on drug procurement and supply are crucial in ensuring adequate and consistent access to essential medicines. These training programs can be aimed towards pharmacists who play an important role in the drug supply cycle [23]. The pharmacy workforce, however, is still lacking and underutilized in many low income countries [23,24].

Third, even when the assessed medications were available, issues surrounding the presence of an antidote or the appropriate storage of the medications were a concern. It is unclear why calcium gluconate, an antidote for MgSO_4_ toxicity, was not stored and whether practitioners were aware of its indication. In addition, oxytocin was not properly stored at a temperature at 2–8°C. This was most likely explained by three factors: (1) lack of refrigerators, (2) pharmacists not aware of the storage condition, and (3) insufficient information printed on package inserts. Provision of the necessary equipment to store medication is extremely important if these medications are to be efficacious when administered to patients. Pharmacists as well as other providers such as physicians, midwives, nurses, and technicians must be aware of the storage conditions for oxytocin. Moreover, drug information labels must be required to provide specific information regarding the storage of medications. In situations where oxytocin is not present or cannot be safely administered by a skilled birth attendant, the WHO EML and WHO guideline for prevention and treatment of PPH recommend the administration of misoprostol, which does not have any storage limitations [10,11]. However, a 2012 survey of 43 countries found that less than one third of the countries had misoprostil available [25].

Last, across all health care professions – physicians, pharmacists, nurses, and midwives – there was a considerable knowledge–practice gap. With oxytocin, even though most health care providers were aware of treatment guidelines, they were not aware of how oxytocin should be used. Continuous education programs and active workshops promoting new standard practice guidelines should be developed and required for all health professionals to enhance hands–on training with these essential medicines.

## LIMITATIONS

A few limitations should be noted in our study. During these rapid assessment exercises, country–specific health facilities were purposefully chosen to minimize constraints due to time limitations, transportation, and human capacity from both the investigators and the local authorities. Therefore, the report findings may not be representative of all health facilities in each country or in all low resource countries. Second, data were extracted from secondary sources (ie, reports), instead of the primary data sources (ie, interview transcripts). Access to the primarily data sources could have provided more information for the Ishikawa diagrams. In addition, the findings of our study may not reflect the most up–to–date use of essential medicines for maternal health in the assessed countries. Lastly, the data were collected at a specific point in time and the barriers and facilitators could vary over time in each country.

Despite these limitations, using the fishbone diagrams as an analytic tool allowed us to identify common barriers and facilitators to the quality use of essential medicines at different health–system levels in seven countries. Follow–up studies could develop and evaluate tailored intervention programs that specifically address these barriers to quality use of these life–saving medicines [26].

## CONCLUSIONS

The “fishbone” Ishikawa diagram is a useful tool for describing the common facilitators and barriers to the quality use of essential medicines for maternal health across countries. The diagram highlighted the complexity between and within each health–system level that must function to ensure the availability, access, and appropriate use of medicines. The specific facilitators and barriers identified should guide the development of tailored intervention programs to improve and expand the use of these life–saving medicines.
