# Supplementary material for: Barriers and facilitators to the quality use of essential medicines for maternal health in low–resource countries: An Ishikawa framework
Source: J Glob Health. 2015 Apr 18;5(1):010406. doi: 10.7189/jogh.05.010406 (PMC4416332; doi:10.7189/jogh.05.010406)

Online Supplementary Document

Tran and Bero. Barriers and facilitators to the quality use of essential medicines for maternal health in low-resource countries: An Ishikawa framework.

J Glob Health 2015;5:010406

Oxytocin Facilitators

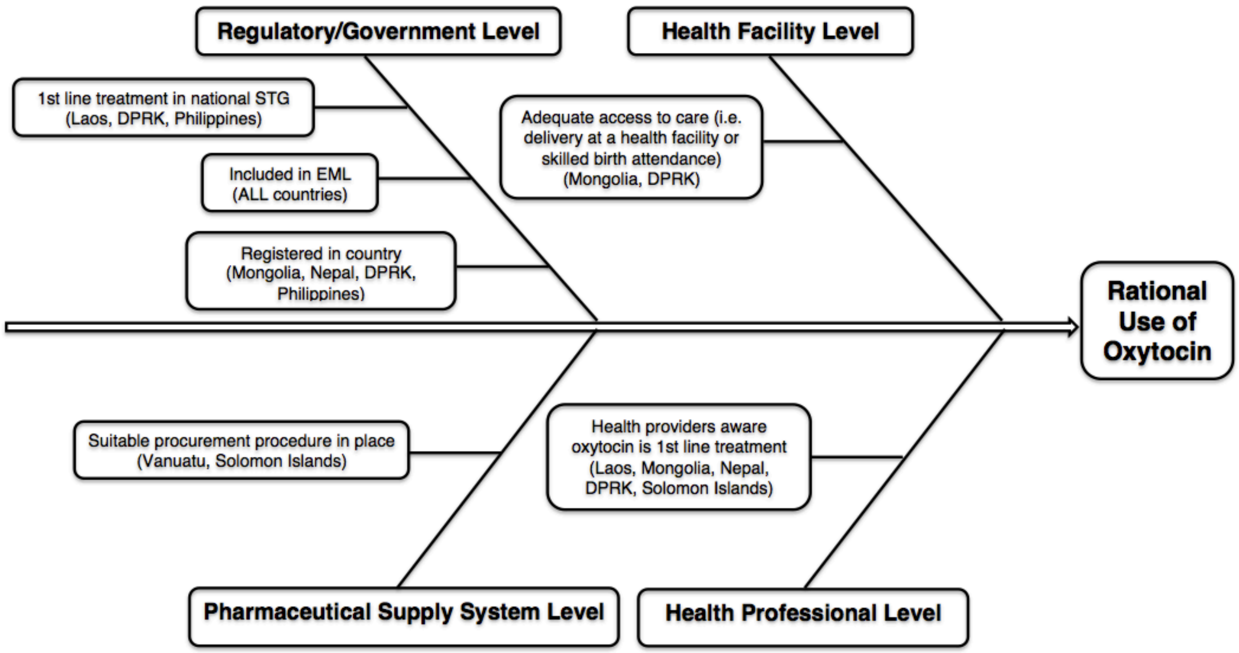

Oxytocin Barriers

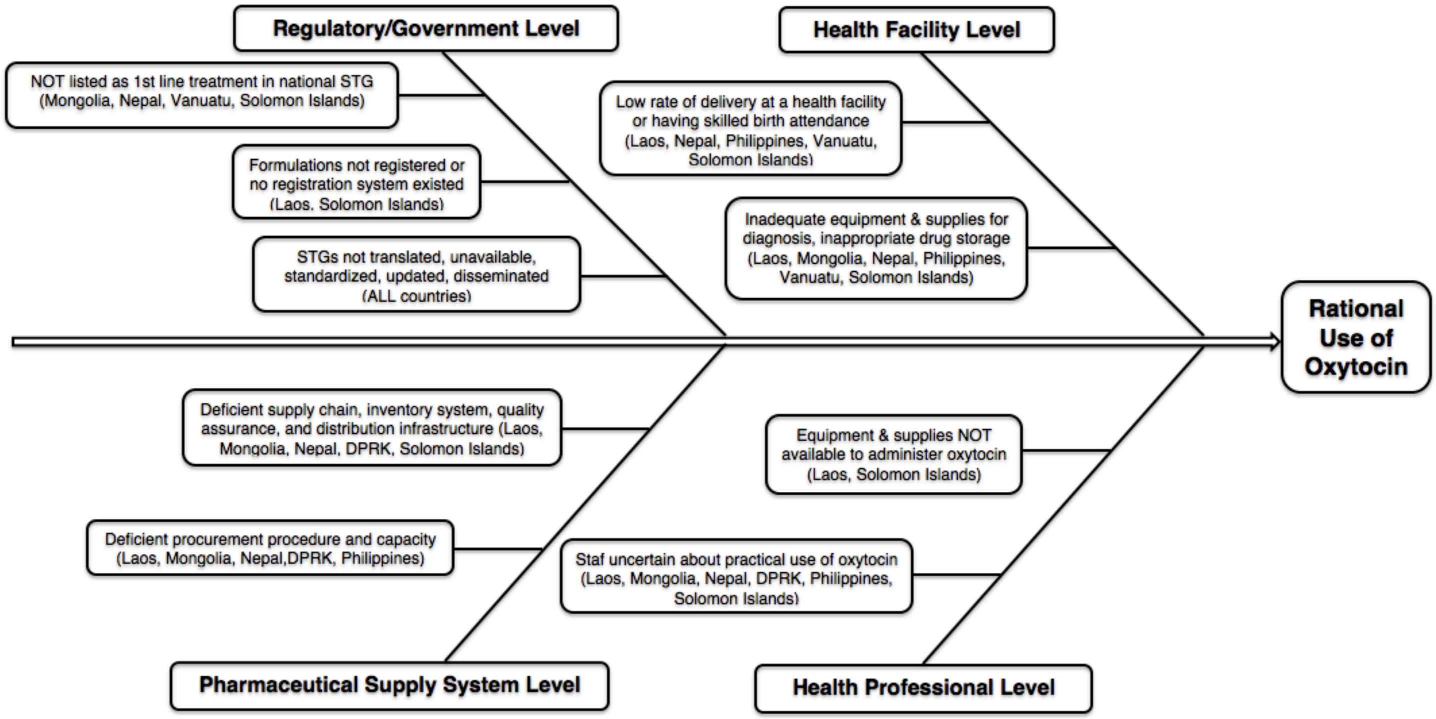

Ergometrine Facilitators

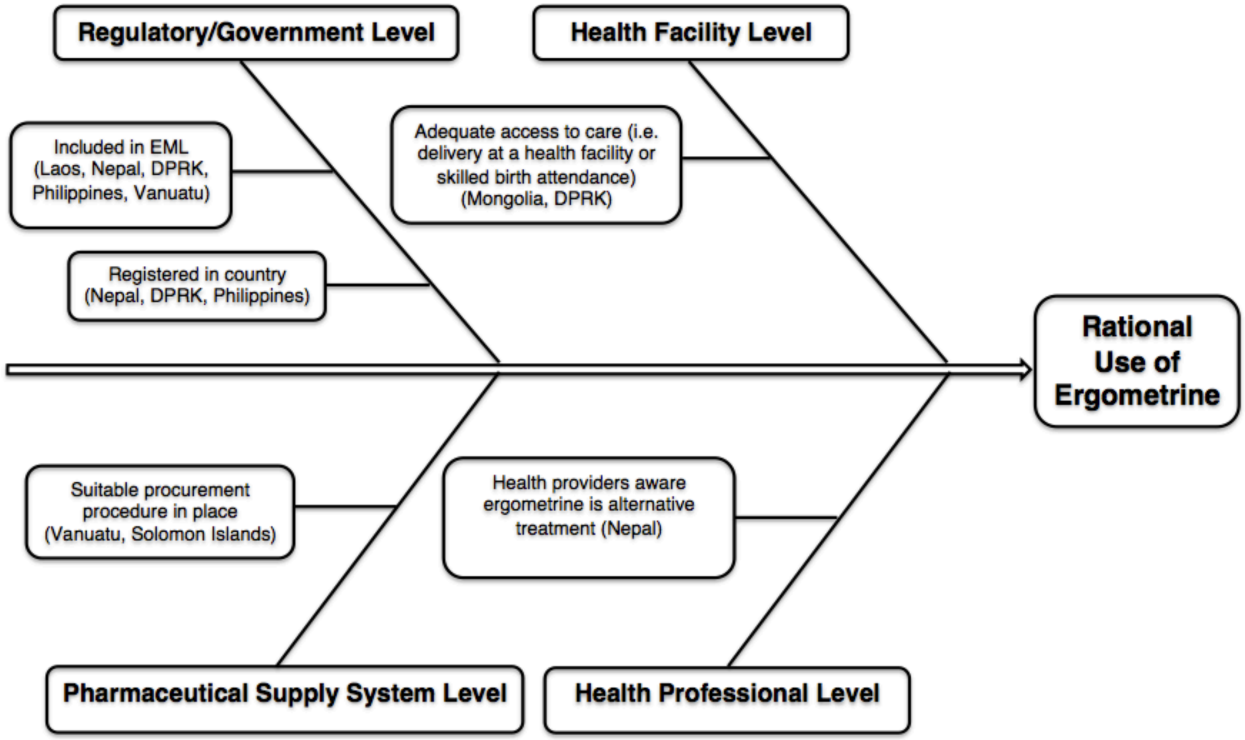

Ergometrine Barriers

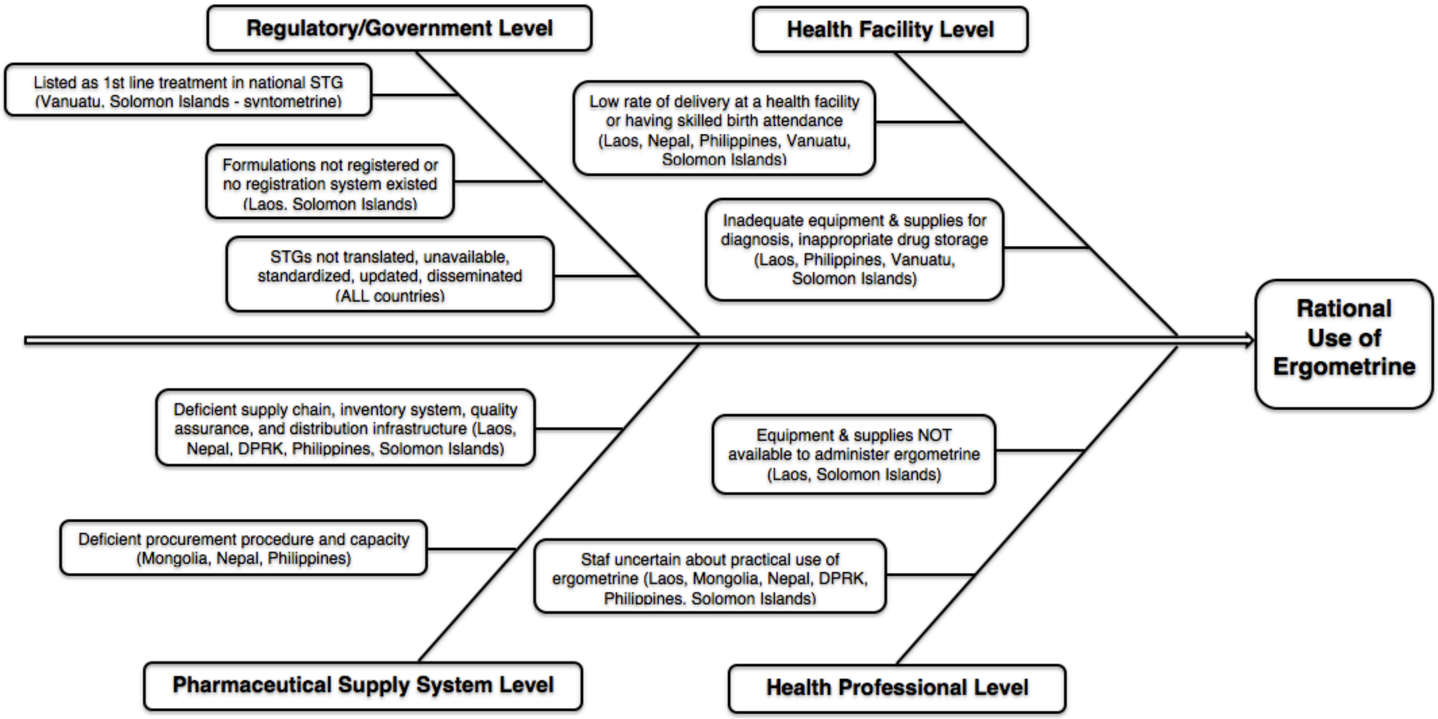

MgSO4 Facilitators

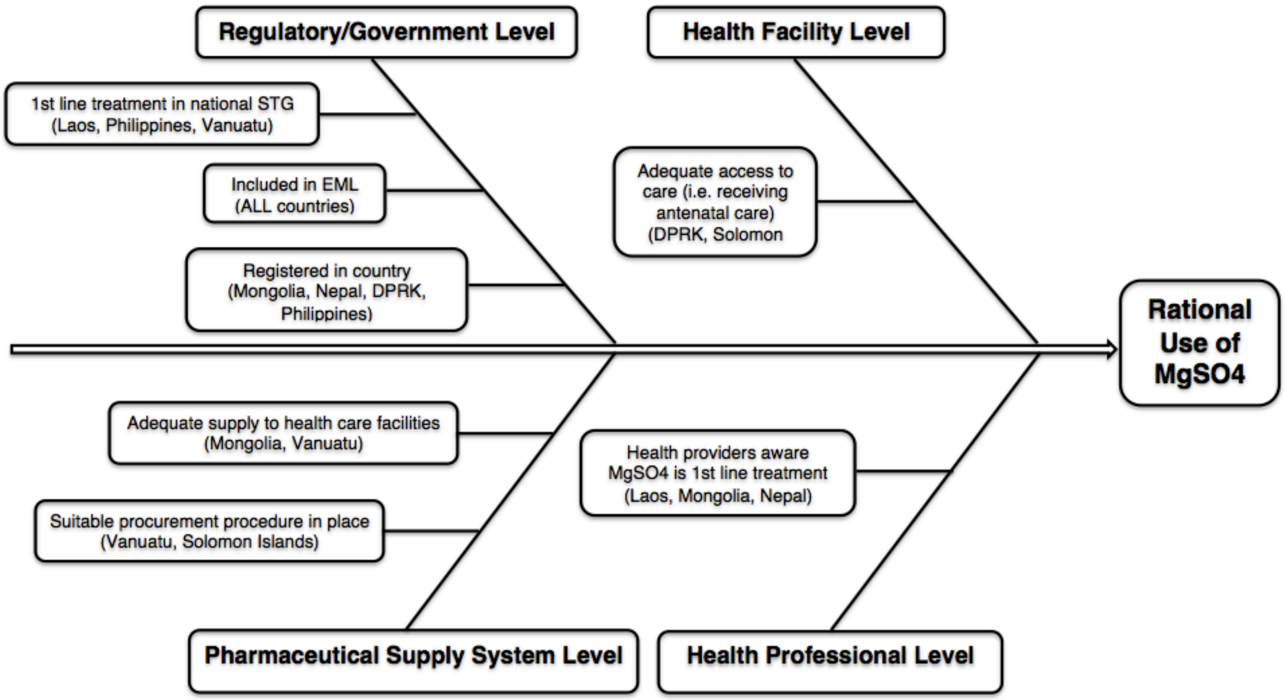

MgSO4 Barriers

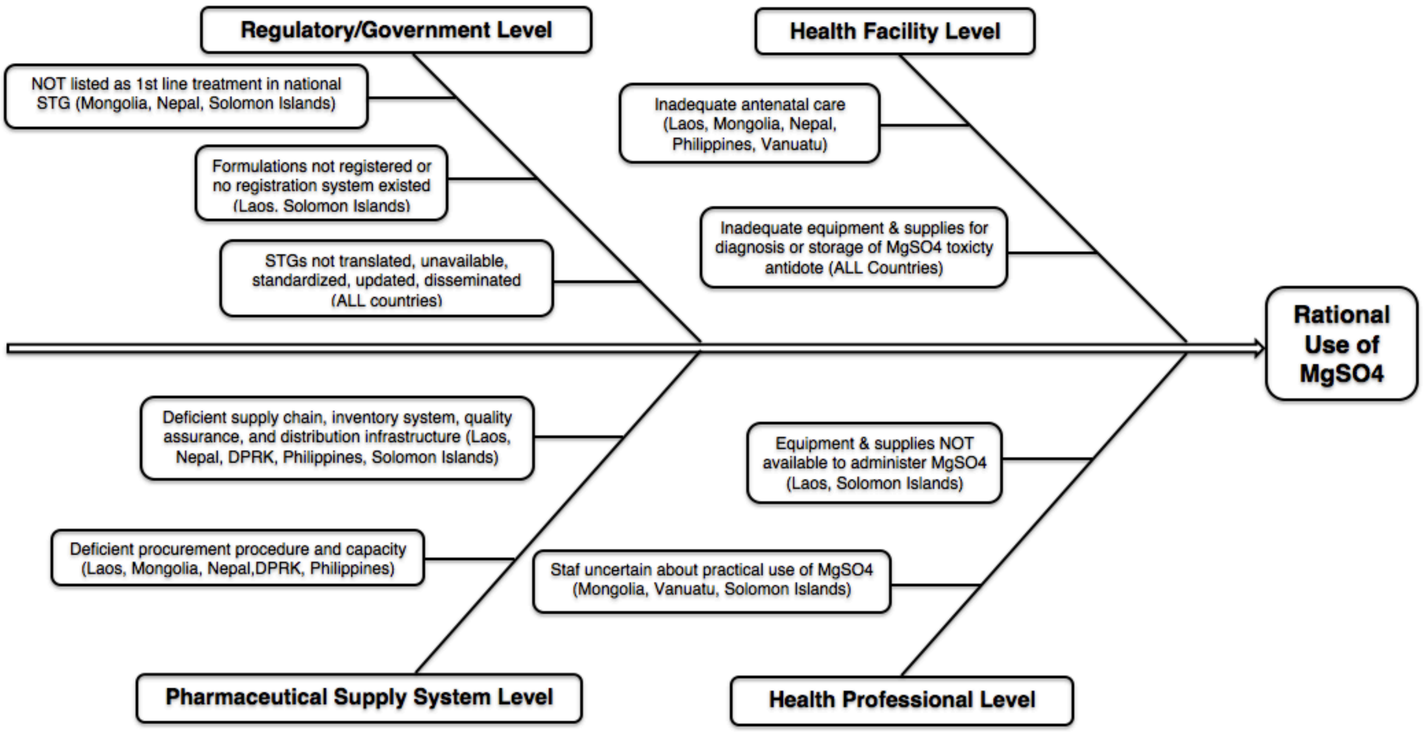

Supplement: Online Supplementary Document [file jogh-05-010406-s001.pdf]
